# Protective Effects of Vitamin D on Proteoglycans of Human Articular Chondrocytes through TGF-β1 Signaling

**DOI:** 10.3390/nu16172991

**Published:** 2024-09-04

**Authors:** Jian Guan, Zhuoxin Li, Guodong Niu, Siwei Li, Weishi Li, Chunli Song, Huijie Leng

**Affiliations:** 1 Department of Orthopedics, Peking University Third Hospital, Beijing 100191, China; guanjian19901008@163.com (J.G.); lzxtxt9916@163.com (Z.L.); tiankun23@163.com (G.N.); lisiwei0831@163.com (S.L.); 2 Department of Orthopedic Surgery, Beijing Luhe Hospital, Capital Medical University, Beijing 101100, China; 3 Engineering Research Center of Bone and Joint Precision Medicine, Ministry of Education, Beijing 100191, China; wslee72@163.com; 4 Beijing Key Lab of Spine Diseases, Beijing 100191, China; schl@bjmu.edu.cn

**Keywords:** osteoarthritis, vitamin D, proteoglycan, TNF-α, TGF-β1 signaling

## Abstract

The extracellular matrix of cartilage primarily constitutes of collagen and aggrecan. Cartilage degradation starts with aggrecan loss in osteoarthritis (OA). Vitamin D (VD) plays an essential role in several inflammation-related diseases and can protect the collagen in cartilage during OA. The present study focused on the role of VD in aggrecan turnover of human articular chondrocytes treated with tumor necrosis factor α (TNF-α) and the possible mechanism. Treatment with different doses of VD and different periods of intervention with TNF-α and TGF-β1 receptor (TGFβR1) inhibitor SB525334 were investigated. The viability of human chondrocytes and extracellular secretion of TGF-β1 were measured. The expression of intracellular TGFβR1 and VD receptor was examined. Transcriptional and translational levels of aggrecan and the related metabolic factors were analyzed. The results showed that TNF-α markedly reduced the viability, TGFβR1 expressions and aggrecan levels of human chondrocytes, and increased disintegrin and metalloproteinase with thrombospondin motifs. The alterations were partially inhibited by VD treatment. Furthermore, the effects of VD were blocked by the TGFβR1 inhibitor SB525334 in TNF-α-treated cells. VD may prevent proteoglycan loss due to TNF-α via TGF-β1 signaling in human chondrocytes.

## 1. Introduction

Osteoarthritis (OA) represents a degenerative condition affecting articular joints, and is mainly characterized by ultimate cartilage degradation without any effective solution for recovery [1,2]. The characteristics of OA also include synovial inflammation, subchondral bone alterations, exacerbation of pain, and reduced mobility [3]. Proteoglycan is widely distributed in all connective tissues and formed of glycosaminoglycans covalently attached to the core proteins [4]. The metabolic balance of proteoglycan and collagen is disturbed in OA. This disease starts with the loss of proteoglycans and collagen [5,6]. By preventing changes in cartilage proteoglycan content in OA, intra-articular hyaluronate can protect against the progression of articular cartilage degeneration [7].

Vitamin D (VD) is a steroidal hormone with diverse biological functions in different tissues, such as the metabolism of bone and cartilage [8]. Several studies have indicated that VD is involved in the regulation of OA, especially cartilage degeneration [9,10]. VD interacts with VD receptors and results in chondrocyte hypertrophy [9]. VD can prevent articular cartilage erosion [10]. Meanwhile, a deficiency of VD increases the risk of OA, which may finally cause collagen loss and cartilage erosion [10]. However, the effects of VD on articular proteoglycan remain unclear.

Since VD is efficient in upholding cartilage integrity during early OA, we hypothesize that VD may protect cartilage through regulating proteoglycans turnover via TGF-β1 signaling in human chondrocytes. We evaluated the effects of VD on the TNF-α-treated chondrocytes and investigated the possible mechanism by examining the cell viability and the protein and mRNA levels of proteoglycan metabolic-associated molecules and TGF-β1 in the human chondrocytes.

## 2. Materials and Methods

### 2.1. Chemicals

The human TNF-α used in the study was from PeproTech Inc. (Rocky Hill, NJ, USA). Calcitrol (VD) and TGF-β1 receptor (TGFβR1) inhibitor were purchased from Selleckchem (Houston, TX, USA). Antibodies of aggrecan, a disintegrin, and metalloproteinase with thrombospondin motifs ADAMTS4, ADAMTS5, and VDR were acquired from Abcam (Cambridge, UK). Additionally, a Cell Counting Kit-8 and human TGF-β1 ELISA kit were also obtained from Abcam.

### 2.2. Cell Culture

Human chondrocytes were procured from Procell science & Technology Co Ltd. (Wuhan, China), along with Dulbecco’s modified Eagle’s medium (DMEM)/F12. The cells were cultured in DMEM/F12 with 10% fetal bovine serum and antibiotics (100 U/mL penicillin and 100 μg/mL streptomycin) at 37 °C in a humidified atmosphere with 5% CO_2_.

### 2.3. Cell Viability Assay

Cell viability was determined by a Cell Counting Kit-8 assay in accordance with the instructions provided by the manufacturer. The cells were seeded at a density of 5 × 10^3^ cells per well into 96-well plates with 100 μL of medium, and then the cells were treated with 100 μL VD (1, 10, 100 nmol/L), or TNF-α (10 ng/mL), or TNF-α (10 ng/mL) and VD (1, 10, 100 nmol/L) for 24 h. The control group was treated with 100 μL medium. A volume of 10 μL of the CCK-8 solution was added to the wells, and after 3 h, the absorbance was measured using a plate reader at 450 nm on an automatic enzyme-linked immunosorbent assay reader (Thermo Fisher Scientific, Waltham, MA, USA). The experiment was conducted with three independent samples per experimental group.

### 2.4. ELISA Analysis

Determination of TGF-β1 release was conducted using an ELISA kit according to the manufacturer’s instructions. The cells were treated with 100 μL of VD (1, 10, 100 nmol/L) or TNF-α (10 ng/mL) or TNF-α (10 ng/mL) and VD (1, 10, 100 nmol/L) for 24 h, while the control group was treated with 100 μL of medium. To determine the total TGF-β1, the samples were initially treated with 1 mol/L HCl for 10 min, followed by neutralization using 1.2 mol/L NaOH/0.5 mol/L HEPES at room temperature. Samples were analyzed directly for active TGF-β1 without prior acid activation. Plates were then read in an automatic enzyme-linked immunosorbent assay reader at a wavelength of 450 nm. The resulting values were then plotted on a standard graph using serial dilution curves provided in the kit.

### 2.5. RNA Extraction and Real-Time Quantitative PCR

The effects of TNF-α (10 ng/mL) on chondrocytes were observed for 4, 8, 12, 24 h. Another series of chondrocytes were treated with 100 μL of TNF-α (10 ng/mL) or a combination of TNF-α (10 ng/mL) and VD (10, 100 nmol/L) for 4, 8, 12, 24 h. The control group was treated with 100 μL of medium. Following lysis of the cell samples in Trizol reagent (Aidlab, Beijing, China), total RNA was extracted, and then reverse transcribed into cDNA using the RevertAid First Stand cDNA Synthesis kit (Thermo Fisher Scientific, Waltham, MA, USA). A volume of 5× Reaction Buffer (4 µL), RNA (1 µg), and RNase-free ddH₂O was combined to make 20 µL of reverse transcription solution in total. The thermocycling conditions were as follows: the reaction was conducted at 25 °C for 5 min, 50 °C for 15 min, 85 °C for 5 min, and 4 °C for 10 min. Relative mRNA expression level was determined by qPCR using a SYBR^®^ Premix Ex Taq™ (Tli RNaseH Plus) PCR kit (Takara Bio, Inc., Kusatsu, Shiga, Japan) and an ABI Prism^®^ 7300 Sequence Detector (Thermo Fisher Scientific, Waltham, MA, USA). GAPDH was employed as the internal control. The relative mRNA level was determined using the 2^−ΔΔCt^ method. The primers were purchased from Thermo Fisher Scientific, Inc. (Waltham, MA, USA). The primer sequences are presented in Table A1.

### 2.6. Immunohistochemistry and Immunofluorescence Analysis

The cells were divided into 5 groups: Control, VD, TNF-α, TNF-α + VD, and TNF-α + VD + SB525334. Immunohistochemistry was performed to demonstrate the presence of VD receptor (VDR) protein. In brief, the cells were fixed with 4% formaldehyde for 15 min, followed by the permeabilization of cells with 0.5% Triton X-100 for 20 min. Subsequently, the cells were incubated with VDR monoclonal antibody overnight at 4 °C, after which they were washed extensively. After this, the cells were incubated with a peroxidase-conjugated secondary antibody for 20 min at room temperature. The color development was facilitated by the use of diaminobenzidine (DAB, 180 μg/mL containing 0.03% hydrogen peroxide). The slides were counterstained with Harris hematoxylin. For immunofluorescence, cells were fixed with 4% formaldehyde for 15 min and permeabilized with 0.5% Triton X-100 for 20 min. Thereafter, the cells were then incubated with the primary antibodies to ADAMTS4, ADAMTS5, and aggrecan overnight at 4 °C, and then washed extensively. After this, cells were incubated with the corresponding fluorescein-isothiocyanate-conjugated secondary antibody (ADAMTS4-FITC, ADAMTS5-FITC, aggrecan-CY3) (BOSTER Biological Technology Co. Ltd., Wuhan, China), and finally evaluated by fluorescence microscopy. Images were captured with an Olympus BX53 biomicroscope (Tokyo, Japan).

### 2.7. Statistical Analysis

Statistical analysis was conducted using GraphPad Prism 5. All the data were expressed as the mean ± standard error of the mean and tested for equality of variance to ensure normal distribution by a Shapiro–Wilk test. Comparisons between multiple groups were using one-way analysis of variance (ANOVA) followed by a Bonferroni post hoc multiple comparison test. A *p*-value of less than 0.05 was considered to indicate a statistically significant result.

## 3. Results

### 3.1. Effects of VD and TNF-α on the Viability of Human Chondrocytes

**Figure 1:**
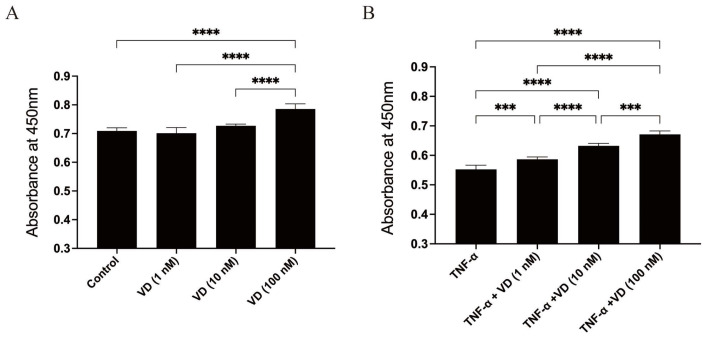
The cell viability of human chondrocytes. (**A**) Comparisons between untreated chondrocytes and chondrocytes with treatment with VD (1, 10, 100 nmol/L). (**B**) Comparisons between untreated chondrocytes and chondrocytes with treatment with TNF-α (10 ng/mL) and/or VD (1, 10, 100 nmol/L). Each bar represents the mean ± SE. Asterisks indicate statistical significance (*** *p* < 0.001, **** *p* < 0.0001).

VD appeared to have increased the viability of human chondrocytes when compared with the cells without any treatment (Figure 1A). As shown in Figure 1B, TNF-α significantly decreased the cell viability, while the treatment with VD significantly hindered the decrease in cell viability due to TNF-α.

### 3.2. Effects of VD and TNF-α on the Release of TGF-β1

**Figure 2:**
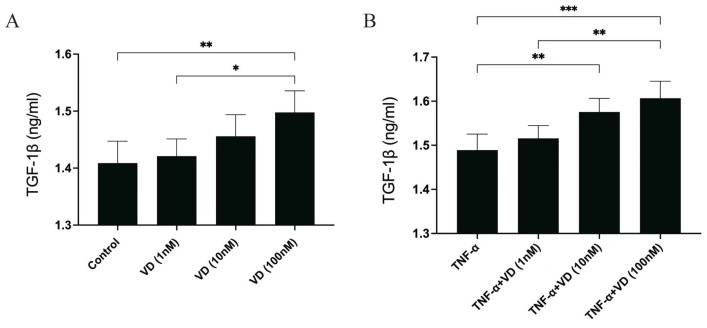
The release of TGF-β1 in human chondrocytes (**A**) after the treatment with VD (1, 10, 100 nmol/L) and (**B**) after the treatment with VD (1, 10, 100 nmol/L) plus TNF-α (10 ng/mL). Each bar represents the mean ± SE. Asterisks indicate statistical significance (* *p* < 0.05, ** *p* < 0.01, *** *p* < 0.001).

The VD treatment demonstrated a trend of increasing the levels of TGF-β1 in the human chondrocytes. Only the highest dose showed a significant difference from the Control group (Figure 2A). Moreover, a high dose of VD could increase TGF-β1 in human chondrocytes treated with TNF-α. (Figure 2B).

### 3.3. Effects of VD on the mRNA Levels of ADAMTS and Aggrecan in TNF-α Treated Human Chondrocytes

**Figure 3:**
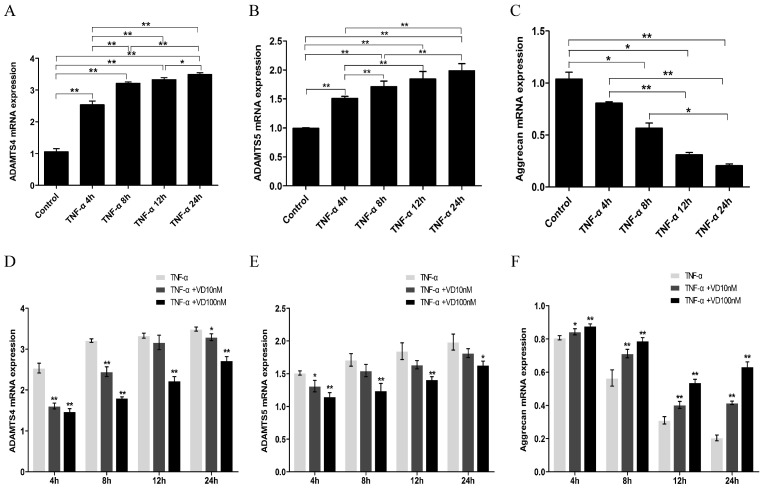
The mRNA expression levels of ADAMTS and aggrecan in human chondrocytes. The mRNA expression level of ADAMTS4 (**A**), ADAMTS5 (**B**), and aggrecan (**C**) in human chondrocytes after the treatment with TNF-α for 4, 8, 12, 24 h. The mRNA expression level of ADAMTS4 (**D**), ADAMTS5 (**E**), and aggrecan (**F**) in human chondrocytes after the treatment with TNF-α (10 ng/mL) plus VD (10, 100 nmol/L) for 4, 8, 12, 24 h. Each bar represents the mean ± SE. Asterisks indicate statistical significance vs. TNF-α group (**D**–**F**) (* *p* < 0.05, ** *p* < 0.01).

Proteoglycans constitute a fundamental component of extracellular matrices, providing structural support and exerting influence over cellular behaviors in both physiological and pathological processes. The secreted metalloprotease ADAMTS4 and ADAMTS5 are responsible for the degradation of cartilage proteoglycan in arthritis [2]. The results showed that TNF-α led to a time-dependent increase in the mRNA levels of ADAMTS4 and ADAMTS5, accompanied by a decrease in the mRNA levels of aggrecan (Figure 3A–C). The changes in the mRNA levels of ADAMTS4, ADAMTS5, and aggrecan were significantly suppressed by VD treatment (Figure 3D–F).

### 3.4. The Role of TGF-β1

**Figure 4:**
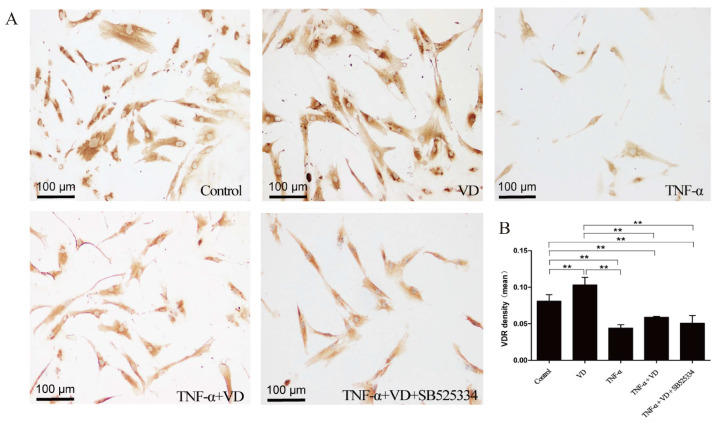
Effects of TGF-β1 downregulation on the expression of VDR. (**A**) The immunohistochemical staining of VDR in human chondrocytes from different groups (scale bar = 100 μm). (**B**) The quantification of VDR density in human chondrocytes from different groups. Each bar represents the mean ± SE. Asterisks indicate statistical significance (** *p* < 0.01).

The role of TGF-β1 was verified by using the TGFβR1 inhibitor SB525334. As shown in Figure 4, the number of VDR-positive cells was significantly increased by VD treatment and decreased by TNF-α treatment. The decrease due to TNF-α was partially inhibited by VD. The decreased activity of TGF-β1 through SB525334 blocked the effect of VD on the number of VDR-positive cells in TNF-α-treated human chondrocytes.

**Figure 5:**
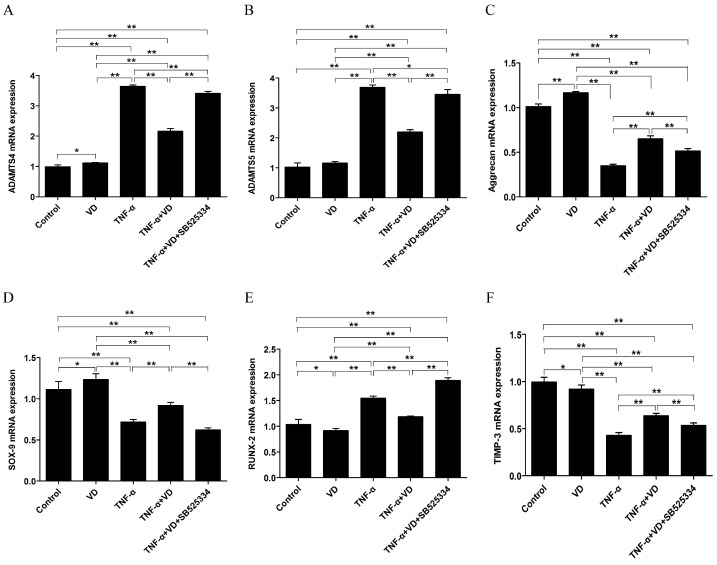
Effects of TGF-β1 activity downregulation on the mRNA levels of proteoglycan metabolic-associated molecules. (**A**) The mRNA expression levels of ADAMTS4 in human chondrocytes. (**B**) The mRNA expression levels of ADAMTS5 in human chondrocytes. (**C**) The mRNA levels of aggrecan in human chondrocytes. (**D**) The mRNA expression levels of SOX-9 in human chondrocytes. (**E**) The mRNA expression levels of RUNX-2 in human chondrocytes. (**F**) The mRNA expression level of TIMP-3 in human chondrocytes. Each bar represents the mean ± SE. Asterisks indicate statistical significance (* *p* < 0.05, ** *p* < 0.01).

**Figure 6:**
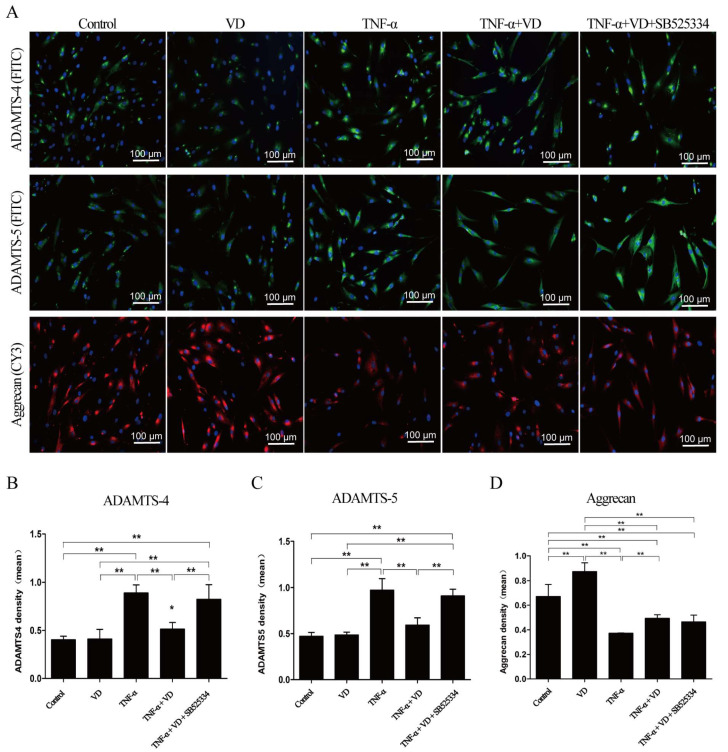
Effects of TGF-β1 activity downregulation on the ADAMTS proteoglycanases and aggrecan. (**A**) The immunofluorescence analysis of ADAMTS4, ADAMTS5, and aggrecan in human chondrocytes (scale bar = 100 μm). FITC-Green; CY3-Red; DAPI-Blue. (**B**) The immunohistochemical quantitative evaluation of ADAMTS4. (**C**) The immunohistochemical quantitative evaluation of ADAMTS5. (**D**) The immunohistochemical quantitative evaluation of aggrecan. Each bar represents the mean ± SE. Asterisks indicate statistical significance (* *p* < 0.05, ** *p* < 0.01).

As shown in Figure 5, VD significantly suppressed the mRNA changes in the proteoglycan metabolism-associated molecules ADAMTS4 (Figure 5A), ADAMTS5 (Figure 5B), aggrecan (Figure 5C), SOX-9 (Figure 5D), RUNX-2 (Figure 5E), and TIMP-3 (Figure 5F) stimulated by TNF-α in the human chondrocytes, which was blocked by the downregulation of TGF-β1 activity. Immunofluorescence analysis also showed that VD significantly suppressed the changes in ADAMTS4 (Figure 6B), ADAMTS5 (Figure 6C), and aggrecan (Figure 6D) induced by TNF-α in human chondrocytes. The downregulation of TGF-β1 activity blocked the effects of VD on the changes in these molecules induced by TNF-α.

## 4. Discussion

VD exerts a profound influence on the condition of numerous articular structures, including cartilage, subchondral bone, and the periarticular muscle, all of which possess a significant function in the advancement of OA [11]. The VD metabolite 24R, 25-dihydroxyvitamin D3 has been demonstrated to provide protection against articular cartilage damage resulting from anterior cruciate ligament transection in male rats [12]. The present study showed that VD may play a role in protection of the proteoglycan integrity on TNF-α-treated human chondrocytes partly through TGF-β signaling.

TNF-α is known as a multifunctional pro-inflammatory cytokine that is involved in a number of pathological processes, including inflammation, immunoregulation, proliferation, and apoptosis [13]. It has been shown that TNF-α enhances the destructive processes in osteoarthritic cartilage [14,15,16]. TNF-α is implicated in the pathogenesis of OA [17,18,19]. The findings revealed that cytotoxicity of TNF-α to human chondrocytes, demonstrated as significantly decreased cell viability, increased the mRNA levels of ADAMTS4 and ADAMTS5, decreased the mRNA level of aggrecan, and decreased the expression of aggrecan.

VD can protect chondrocytes from damage in multiple ways. The results showed that VD treatment can decrease the levels of TNF-α, reduce the cytotoxicity of TNF-α, and increase cell viability, VDR and TGF-β expressions of human chondrocytes. In the literature, treatment with VD was also reported to be able to reduce the levels of TNF-α [20]. Kong’s study also indicated that active VD treatment was effective in preserving chondrocyte viability [20]. VD can act by relying on vitamin D receptor (VDR) binding. Interestingly, VD treatment can also increase VDR expression of chondrocytes. Positive and significant correlations were also observed between VD levels and VDR expression in other studies [21,22]. TGF-β was suggested to play a significant role in cartilage metabolism. Our preceding experiments showed that 1α,25(OH)_2_D_3_ increased the expression of TGF-β1 in TNF-α-stimulated rat chondrocytes in a dose-dependent manner [10].

The changes in mRNA in ADAMTS4, ADAMTS5, and aggrecan were significantly suppressed by VD treatment. The progression of OA is accompanied by a shift in the equilibrium between the synthesis and the degradation of the cartilage matrix, culminating in a gradual decline in articular cartilage [23]. Proteoglycans, mainly as aggrecan in articular cartilage, are responsible for providing the compressive resistance to cartilage at the joints [24]. In a human model of OA, the inhibition of ADAMTS4 and ADAMTS expression in human cartilage effectively reduces the loss of aggrecan [25]. Aggrecan is one of the first extracellular matrix components to undergo measurable loss in arthritic diseases, and plays a protective role in preventing the degradation of collagen fibrils. An aggrecans inhibitor may impart overall cartilage protection [26]. The ADAMTS4 level was found to be significantly elevated during the early stages of OA when compared to the intermediate and advanced stages of OA, as well as in healthy control subjects [27]. The present study provided evidence that VD might be able to prevent early proteoglycan loss as an ADAMTS inhibitor.

There is still debate among the scientific community regarding the specific role of the TGF-β pathway in the pathogenesis of OA [28,29]. Some studies found that the inactivation of TGF-β or its downstream molecules may represent a crucial signaling event contributing to the pathogenesis of OA [30]. Additionally, it was observed that TNF-α exerted a suppressive effect on TGF-β activity in both human OA and bovine articular chondrocytes [31]. Our results demonstrated that VD increased the secretion of TGF-β1 in human chondrocytes and those treated with TNF-α, suggesting that TGF-β1 may be implicated in the pathogenesis of TNF-α induced cytotoxicity in human chondrocytes. Furthermore, VD may confer protection to TNF-α treated human chondrocytes by enhancing TGF-β1 secretion.

It has been reported that TGF-β1 can stimulate articular chondrocyte proteoglycan synthesis in the murine knee [32]. Our previous study demonstrated that VD can prevent articular cartilage erosion through upregulating the expression of TGF-β1 in ovariectomized rats. Members of the TGF-β family play critical roles in the metabolism and differentiation of articular chondrocytes, and have been linked to the pathological mechanisms of OA [14]. The secretion of TGF-β1 was found to be increased by VD in TNF-α treated human chondrocytes and the control cells. Moreover, downregulation of TGF-β1 activity with the treatment of TGFβR1 inhibitor SB525334 blocked the effect of VD on the changes in ADAMTS4, ADAMTS5, aggrecan, SOX-9, RUNX-2, and TIMP-3 induced by TNF-α in human chondrocytes demonstrated by both real-time PCR and the immunofluorescence analysis. Thus, considering VD’s protective roles on the TNF-α treated human chondrocytes by regulating the proteoglycans turnover, VD is a potential nutrition and treatment strategies for osteoarthritis.

Regarding the limitations, the present study was based on experiments at the cellular level which utilized the normal chondrocytes treated by TNF-α, and could not reflect the condition of VD deficiency. In the future, we may design cell experiments using primary chondrocytes from VD-deficient animals to further confirm the effects of VD treatment on VD-deficient chondrocytes. Animal in vivo studies have already shown that VD deficiency can lead to cartilage degeneration. It is reasonable that VD supplementation might help cartilage recovery for OA patients with or without VD deficiency. Further clinical studies investigating the correlation between VD intake and reduced incidence and severity of OA may shed light on VD’s implication for a new clinical strategy.

## 5. Conclusions

In conclusion, our study revealed that VD can protect human chondrocytes against TNF-α induced cytotoxicity and promote the anabolism of proteoglycans and the release of TGF-β1 in TNF-α treated human chondrocytes. The increased TGF-β1 may serve as an intermediary in the metabolism of proteoglycans in TNF-α treated human chondrocytes. VD may represent a promising therapeutic avenue in OA.

## Data Availability

Data are contained within the article, further inquiries can be directed to the corresponding authors.

## Appendix A

**Table A1:** Primer sequences.

Names	Primers	Sequence
GAPDH	Forward	5′-TCAAGAAGGTGGTGAAGCAGG-3′
	Reverse	5′-TCAAAGGTGGAGGAGTGGGT-3′
Aggrecan	Forward	5′-TGAGCGGCAGCACTTTGAC-3′
	Reverse	5′-TGAGTACAGGAGGCTTGAGG-3′
ADAMTS-4	Forward	5′- CAATCCTGTCAGCTTGGTGG-3′
	Reverse	5′-GCTGTGTCAAAGTGGTCAGG-3′
ADAMTS-5	Forward	5′-CTGCCACCACACTCAAGAAC-3′
	Reverse	5′-TGGAGGCCATCGTCTTCAAT-3′
SOX-9	Forward	5′-GAACGCACATCAAGACGGAG-3′
	Reverse	5′-AGTTCTGGTGGTCGGTGTAG-3′
RUNX-2	Forward	5′-ATTCTGCTGAGCTCCGGAAT-3′
	Reverse	5′-AGCTTCTGTCTGTGCCTTCT-3′
TIMP-3	Forward	5′-GTCGCGTCTATGATGGCAAG-3′
	Reverse	5′-AAGCAAGGCAGGTAGTAGCA-3′
